# Personality functioning in adolescents and its association with health-related quality of life and physical fitness

**DOI:** 10.3934/publichealth.2025047

**Published:** 2025-09-12

**Authors:** Vera Prünster, Kirstin Goth, Martin Niedermeier, Klaus Greier, Karin Labek, Gerhard Ruedl

**Affiliations:** 1 Department of Sport Science, University of Innsbruck, 6020 Innsbruck, Austria; 2 Department of Child and Adolescent Psychiatry, University Clinics Saarland, 66421 Homburg, Germany; 3 Institute of Psychology, University of Innsbruck, 6020 Innsbruck, Austria

**Keywords:** level of personality functioning, health-related quality of life, physical fitness, well-being, youth, identity, self-direction, empathy, intimacy

## Abstract

Adolescence is a crucial developmental phase marked by major physical, cognitive, and psychosocial changes that shape self-perception and relationships, with lasting effects on mental and physical health. Personality functioning, a core concept in modern diagnostic systems, such as the International Classification of Diseases (ICD-11), offers a dimensional framework that incorporates key developmental domains such as identity, self-direction, empathy, and intimacy. Early detection of impairments in these areas is essential to promote mental and physical well-being and to prevent the onset of mental disorders. In addition, an improvement in physical fitness (PF) appears to be associated with a significantly lower risk of developing mental disorders. Thus, this study aimed to examine associations between the dimensions of personality functioning, health-related quality of life (HRQoL), and PF in adolescents. A total of 186 adolescents (48.3% girls; mean age 15.6 ± 0.6 years) completed the KIDSCREEN-10, the Levels of Personality Functioning Questionnaire (LoPF-Q) 12–18, and the standardized German motor fitness test (DMT 6–18). Significant negative moderate and large correlations were found between HRQoL and overall personality dysfunction, as well as the identity, self-direction, empathy, and intimacy development domains. Additionally, PF showed significant negative moderate correlations with overall personality dysfunction, particularly with intimacy. Multiple regression analyses revealed that self-direction and intimacy were significantly negatively associated with the HRQoL, while intimacy was negatively associated with PF. Impaired personality functioning, particularly in self-direction and intimacy, was strongly associated with reduced HRQoL and PF in adolescents. These findings highlight the importance of recognizing emerging personality difficulties early and providing timely support, as this can play a vital role in promoting both mental and physical health during adolescence and later in life.

## 1. Introduction

Adolescence represents a critical developmental phase, marked not only by significant biological changes but also by a range of psychosocial transitions [1]. During this period, psychological risk factors, such as depressive symptoms, anxiety, and low self-esteem, frequently emerge, potentially leading to profound implications for both physical and mental health [2]. Mental health risk factors in adolescence are frequently linked to an elevated risk of chronic diseases in adulthood, thus underscoring the importance of prevention and intervention strategies during this vulnerable stage of life [1]. Various classification systems for the diagnosis and classification of mental disorders and diseases, such as the Diagnostic and Statistical Manual of Mental Illnesses (DSM-5) [3], the Alternative Model of Personality Disorders (AMPD), and the International Classification of Diseases (ICD-11) [4], emphasize the importance of personality functioning in assessing psychopathology, including in adolescents.

These models describe personality functioning through core dimensions that are crucial for mental health and interpersonal relationships [5]. Identity, self-direction, empathy, and intimacy are the central dimensions that play pivotal roles in shaping mental health [5]. Impairments in these areas can lead to identity uncertainty, impulsive behaviors, lack of empathy, and unstable relationships, which are key characteristics of personality disorders in the DSM-5, AMPD, and ICD-11 [5]. In this context, Goth et al. [6] emphasized the significance of early identification of personality dysfunctions in adolescents, and Zimmermann et al. [5] argued that a nuanced assessment of developmental risks in personality can be achieved through the consideration of the four essential dimensions – identity, self-direction, empathy, and intimacy.

Identity refers to the stability of one's self-image. Self-direction involves the ability to pursue goals and regulate oneself. Empathy is the capacity to understand others' emotions. Intimacy/attachment pertains to forming close, trusting relationships [7]. When a disturbance of self-image is present, the affected individuals often experience frequent and drastic shifts in their self-perception [8]. Additionally, there may be inconsistent or contradictory views about oneself depending on the current mood or social context. An unstable self-image can make it difficult to pursue long-term goals, as individuals may struggle with fluctuating self-perception [8]. Uncertainty regarding one's identity can lead to problems in interpersonal relationships, as individuals may struggle to act authentically or establish stable connections [8]. These symptoms are characteristics of mental disorders such as borderline personality disorder [8].

A disturbance in the ability for self-direction and goal pursuit is characterized by impulsivity, which leads individuals to often act spontaneously without considering consequences, a lack of goal orientation, which makes it difficult to set and pursue clear objectives, emotional instability, particularly mood swings and increased irritability, and organizational problems, which manifests as the inability to structure tasks and effectively prioritize [9]. Such impairments are typical of attention deficit hyperactivity disorder (ADHD), a disorder that can significantly affect both academic and social functioning [9].

A disturbance in empathy occurs when individuals struggle to recognize or understand others' emotions, which leads to social difficulties and possible conflicts [10]. A lack of empathy can lead to social isolation or difficulties in forming meaningful relationships [10]. For example, these impairments are characteristic of narcissistic personality disorder [10].

An impairment of intimacy can manifest as attachment disorders, which may be present when there is a disturbance in the ability to develop and maintain close, trusting relationships. This disorder is characterized by emotional distance, lack of trust, and instability in relationships [11]. In this context, Hartmann et al. [12] examined the impact of social relationships on adolescent personality, and found that poor social integration within peer groups significantly heightened the risk for impaired personality development. These findings highlight the pivotal role of social factors in educational settings for prevention and intervention efforts.

Research increasingly highlights the significance of personality functioning for the overall quality of life and physical fitness (PF), thereby suggesting a reciprocal relationship between mental state, daily functioning, and athletic performance across all age groups [13]. Personality functioning, as described in the DSM-5, AMPD, and ICD-11, plays a crucial role in shaping an individual's ability to maintain a stable sense of self, regulate emotions, and establish meaningful relationships—all of which contribute to overall life satisfaction and well-being [14]. Individuals with impairments in personality functioning often experience heightened psychological distress, difficulties in interpersonal interactions, and a reduced adaptability to life challenges, which lead to a diminished quality of life [5]. In contrast, a well-integrated personality, characterized by self-direction, emotional stability, and social competence, is associated with greater life satisfaction, stronger resilience to stress, and an improved psychological well-being, as deduced in a study with 12 to 20 years old participants [15]. Therefore, fostering healthy personality development, particularly in adolescents, is crucial to improve both mental well-being and overall quality of life in youth [16].

The relationship between personality functioning and PF is equally noteworthy, as growing evidence suggests that enhanced PF is associated with lower incidences of mental disorders in adolescence and adulthood [17]. According to a study among adolescents aged 13–14 by Åvitsland et al. [18], higher PF, as measured by cardiorespiratory fitness, is significantly associated with lower levels of psychological distress. In their longitudinal study from childhood to adolescence, Chiang et al. [19] further demonstrated that higher PF is linked to a significantly reduced risk of developing mental disorders. Given that individuals with impaired personality functioning often struggle with motivation, self-discipline, and adaptive coping mechanisms, they may be less likely to engage in regular physical activity, thereby increasing their vulnerability to both physical and mental health issues [20],[21]. Conversely, young adults with higher levels of self-regulation and emotional stability (i.e., key components of personality functioning) are more likely to adopt health-promoting behaviors, which includes regular exercise [22]. This is regarded as fostering greater resilience to psychological distress and contributes to overall well-being [23].

While the aforementioned studies addressed personality functioning, they did not establish a connection with health-related quality of life (HRQoL) or PF. While HRQoL refers to the subjective perception of one's physical, psychological, and social well-being in relation to health, PF includes, among other components, cardiorespiratory endurance, muscular strength, speed, and flexibility [24],[25]. This is particularly relevant during adolescence, as PF is closely linked to both physical and psychological development in this stage of life [18]. These concepts are of central importance for adolescents, as they are in a formative phase during which lifestyle habits are established and the foundation for future health is laid [17]. Good PF can not only reduce the risk of chronic diseases, but is also associated with improved mental health, academic performance, and social participation [19]. In turn, HRQoL provides a comprehensive measure to capture subjective well-being and life satisfaction [16]. To the best of our knowledge, no previous study has systematically explored the complex interplay between personality functioning, HRQoL, and PF in adolescents. Thus, the present study aims to address this gap by examining how distinct dimensions of personality functioning – namely identity, self-regulation, empathy, and intimacy – are associated with an adolescent's perceived quality of life and their PF levels. In line with existing research, we hypothesized the following: a) negative associations between the dimensions of personality functioning and quality of life; and b) negative associations between the dimensions of personality functioning and PF.

## 2. Materials and methods

### 2.1. Study design and participants

A cross-sectional design was employed, and 5 secondary grammar schools from the Austrian federal state of Tyrol, where direct contact with the school administration was possible, were invited to participate using convenience sampling. To ensure that the curriculum schedules were similar across participants, only grammar schools were included. The inclusion criteria for participants were as follows: a) enrollment in the 10th grade of a public grammar school; and b) provision of informed consent. Data collection took place between April and June 2023. A total of 186 adolescents (54.8% girls) with a mean age of 14.31 ± 0.51years participated in the study.

Before data collection, approval for the surveys and fitness assessments was obtained from the Tyrolean Education Directorate, the Ethics Advisory Board of the University of Innsbruck (Certificate of Good Standing, 73/2021), and the school principals.

### 2.2. Test procedures

#### 2.2.1. Level of personality functioning

The Levels of Personality Functioning Questionnaire (LoPF-Q) 12–18 is a self-report questionnaire designed to measure impairment in personality functioning in adolescents aged 12 to 18 years. The questionnaire was developed by Goth et al. [6] based on the criteria of the DSM-5, AMPD, and the ICD-11. The questionnaire consists of 97 items that focus on identity, self-direction, empathy, and intimacy [6]. The items are assessed using a 5-point scale, ranging from 0 (no) to 4 (yes). The questionnaire provides a total score alongside four dimension scores, each corresponding to one aspect of Criterion A, which refers to the core impairment in self and interpersonal functioning [26]: identity (e.g., “I am unsure of what kind of person I truly am”), self-direction (e.g., “I struggle to achieve the goals I set for myself”), empathy (e.g., “I often have trouble understanding how others react to my behavior”), or intimacy (e.g., “I prefer not to have others get too close to me”). For each scale, a T-score is calculated based on the responses to the corresponding items [27]. Then, a global personality functioning score is derived from these scales to assess the overall personality functioning across the instrument's various levels [27]. Since the focus is on identifying impairments in personality functions, below-average scores indicate healthy, mature functioning, and average scores reflect age-appropriate development. If two or more personality functions are significantly above average (*T*-scores above 60 = markedly impaired, *T*-scores above 70 = severely impaired), then there is an increased risk of a developing or existing personality disorder [27]. Therefore, a more in-depth diagnostic evaluation is recommended [27].

Cronbach's alpha values indicated high internal consistency: the total scale showed a scale reliability of 0.97, with 0.92 for identity (23 items), 0.94 for self-direction (25 items), 0.87 for empathy (26 items), and 0.92 for intimacy (23 items) [6]. The validity of the questionnaire was confirmed through significant differences between a school sample and a patient group diagnosed with personality disorders (SKID-II), with effect sizes ranging from d = 2.0 to 2.2 standard deviations [27]. The results demonstrate the high reliability and validity of the questionnaire, which supports its use for the early identification of personality disorders in adolescence.

#### 2.2.2. Health-related quality of life

The HRQoL was assessed using the KIDSCREEN-10 questionnaire, which is a psychometrically robust and validated instrument designed to evaluate the HRQoL in youth populations [24]. This tool is intended as a screening, monitoring, and assessment measure for children and adolescents aged 8–18, regardless of the presence of chronic conditions [28]. The reliability and validity of the KIDSCREEN-10 have been rigorously assessed and confirmed [29]. In addition to its good internal consistency (Cronbach's alpha = 0.82), the index also demonstrates good test-retest reliability (*r* = 0.73; ICC = 0.72), which indicates that it is a precise and stable instrument to measure the HRQoL [24]. The KIDSCREEN-10 index offers a composite measure of quality of life, thereby aencompassing physical and psychological well-being, interpersonal relationships with parents and peers, and satisfaction with school life [28]. It consists of 10 items, each rated on a 5-point Likert scale ranging from 1 (“not at all”) to 5 (“extremely”), with higher scores reflecting a better HRQoL [28]. The item scores are summed to create a total T-score, which is then transformed using RASCH-Person parameter estimates into *T*-scores [30].

The participants whose *T*-scores exceeded the sex-specific European normative mean values — 49.00 for females and 51.12 for males, as established by Ravens-Sieberer et al. [30] — were categorized as having a high HRQoL. *T*-scores above this threshold indicate high psychological well-being, characterized by descriptors such as being happy, viewing life positively, being satisfied with life, and being emotionally balanced [30]. In contrast, lower scores reflect a diminished psychological well-being, described as “no joy in life, feeling depressed, feeling unhappy, and having low self-esteem” [30].

#### 2.2.3. Physical fitness

For the 6–18 age group, PF was evaluated using the German Motor Performance Test [31], which is a standardized series consisting of eight distinct components designed to assess various dimensions of PF. The test included the following tasks: a 20-meter sprint to measure sprint speed, backward balancing on three 3-meter-long beams of varying widths to evaluate coordination and precision, side-to-side jumps across a central line for 15 seconds to assess coordination under time constraints, the stand-and-reach test to measure flexibility, push-ups and sit-ups performed over 40 seconds to assess strength endurance, the standing long jump to measure explosive power, and a 6-minute run to evaluate aerobic endurance [25]. According to Bös et al. [25], the series demonstrated high inter-rater reliability (0.95) and satisfactory test-retest reliability (0.82), and has been validated to measure speed, coordination, flexibility, strength, and endurance. The assessments were conducted in the high schools of the participating schools by physical education (PE) students who had undergone specialized training. All procedures were rigorously followed in accordance with the test manual by Bös et al. [25].

The results from the test items were standardized using age- and sex-specific reference values, with a *Z*-score of 100 representing the average performance for each test [31]. Z-scores exceeding 100 indicated above-average performance, while scores below 100 reflected below-average performance. The mean of all standardized *Z*-scores was calculated to provide a comprehensive measure of overall PF, referred to as the total *Z*-score [31].

### 2.3. Statistics

The statistical analyses were performed using IBM SPSS software, version 29.0.0.0. Descriptive statistics are presented as means ± standard deviations, alongside both absolute and relative frequencies. Normal distribution was tested via the Shapiro-Wilk procedure. Differences in continuous variables (identity, self-direction, empathy, intimacy, HRQoL, and PF) were assessed using either independent t-tests or Mann–Whitney U tests, depending on the distribution of the data. Furthermore, correlations between the total LoPF, identity, self-direction, empathy, intimacy, HRQoL, and PF were calculated. Depending on the distribution of the data, either Pearson's product-moment correlation coefficient or Spearman's rank correlation coefficient was used.

Following Cohen's [32] guidelines, the effect size of the correlations were categorized as small to moderate (*r* = 0.1 to 0.3), moderate to large (*r* = 0.3 to 0.5), and large (*r* > 0.5). Variables with a p-value of less than 0.1 for the correlation coefficients were entered into two separate multiple linear regression analyses [33], one analysis with the HRQoL and the other analysis with PF as the dependent variable. Furthermore, the regression analyses were adjusted for sex due to the demonstrated sex-specific differences in the HRQoL [30] and PF [19]. Multicollinearity and autocorrelation of the residuals were examined in advance, with a variance inflation factor (VIF) above 5 considered critical, and a Durbin-Watson value~2 considered as no autocorrelation, <2 as a positive autocorrelation, and >2 as a negative autocorrelation [34]. According to Cohen's [32] interpretation of *R^2^*, an *R^2^* value of 0.02 indicates a small amount of explained variance, an *R^2^* value of 0.13 indicates a medium amount of explained variance, and an R² value of 0.26 or higher indicates a large amount of explained variance. All *p*-values were two-tailed, with values below 0.05 considered statistically significant.

## 3. Results

In this school sample of 186 adolescents, the mean *T*-score for the HRQoL was 49.04 ± 9.54 (girls: 47.00 ± 8.08; boys: 51.45 ± 10.57) and the mean total Z-score for PF was 103.05 ± 8.21. The participants had a mean Body-Mass-Index of 20.78 ± 3.34 kg/m^2^ (girls: 21.04 ± 3.22 kg/m^2^; boys: 20.46 ± 3.47 kg/m^2^). Table 1 presents the absolute values and the Z standardized values of the fitness test.

**Table 1.** Means ± standard deviations of the *Z*-scores and absolute values of the fitness test.

Test-item	Mean *Z*-value ± *sd*	Absolute mean value ± *sd*
Sprint	107.46 ± 12.12	3.64 ± 0.42 seconds
Balancing backwards	100.69 ± 12.20	34.91 ± 11.28 steps
Jumping sideways	108.94 ± 14.67	39.66 ± 12.71 repetitions
Trunk bend forward	104.13 ± 11.52	3.52 ± 10.08 cm
Push-ups	105.46 ± 13.98	15.75 ± 7.43 repetitions
Sit-ups	94.91 ± 10.07	23.97 ± 7.43 repetitions
Standing long jump	103.87 ± 12.19	172.97 ± 44.71 cm
6 minutes run	99.67 ± 15.19	1055.97 ± 384.81 m

Note: In sprint tests, lower absolute values are associated with better performance. Positive absolute values in forward trunk bending are indicative of higher flexibility. For all other tests, higher absolute values are associated with better performance.

Table 2 presents the mean values for the LoPF *T*-scores and the threshold values for impairment in the LoPF. Regarding the total LoPF score, about 26% of adolescents showed an increased risk developing a personality disorder (*T* scores > 60) and about 5% had an existing personality disorder (*T* scores > 70).

**Table 2.** Means ± standard deviations of the LoPF total *T*-score and of the *T*-scores for the four dimensions of the LoPF, as well as the numbers (percentages) of *T* scores > 60 and >70.

	mean LoPF total *T*-score	mean identity *T*-score	mean self-direction *T*-score	mean empathy *T*-score	mean intimacy *T*-score
	51.15 ± 11.48	51.52 ± 11.44	51.03 ± 11.50	48.87 ± 11.68	52.97 ± 11.00
*T*-scores >60	*N* = 48 (25.8%)	*N* = 49 (26.3%)	*N* = 38 (20.4%)	*N* = 35 (18.8%)	*N* = 51 (27.5%)
*T*-scores >70	*N* = 9 (4.8%)	*N* = 9 (4.8%)	*N* = 13 (7.0%)	*N* = 7 (3.8%)	*N* = 12 (6.5%)

Note: LoPF = Level of Personality Functioning.

Table 3 shows the correlation between the values of the total LoPF-Q and the four subdimension scores (identity, self-direction, empathy, intimacy) with HRQoL, as well as with PF. All correlations yielded significant values, except for the correlation between PF and self-direction. Higher values for both the HRQoL and PF were associated with lower values for impairments in personality functioning, all speaking for a healthy development.

**Table 3.** Spearman correlation (*r*) between health-related quality of life and physical fitness, and LoPF total *T*-value and the four scales of LoPF *T*-values (*N* = 186).

	Health-related quality of life		Physical fitness	
	*r*	*p*-value	*r*	*p*-value
LoPF total *T*-value	−0.55	<0.001*	−0.21	0.005*
LoPF identity *T*-value	−0.49	<0.001*	−0.16	0.028*
LoPF self-direction *T*-value	−0.52	<0.001*	−0.12	0.117
LoPF empathy *T*-value	−0.44	<0.001*	−0.16	0.027*
LoPF intimacy *T*-value	−0.51	<0.001*	−0.24	0.001*

Note: * *p* < 0.05, LoPF = Level of Personality Functioning.

Table 4 presents the results of the multiple linear regression analysis with the HRQoL as the dependent variable. The four LoPF dimensions explained 30.2% of the variance of the HRQoL, and the model was significant with *p* < 0.001. The tests on the assumptions did not indicate multicollinearity of the predictor's identity (VIF = 3.74, *T* = 0.27), self-direction (VIF = 3.59, *T* = 0.28), empathy (VIF = 2.04, *T* = 0.49), and intimacy (VIF = 2.29, *T* = 0.44), nor an autocorrelation of the residuals, with a Durbin-Watson value of 1.90. Of the four dimensions, self-direction and intimacy showed significant negative associations with the HRQoL, thus indicating that high values of the HRQoL were linked with an impairment in personality functioning.

**Table 4.** Results of the multiple linear regression analysis on the dependent variable health-related quality of life (*N* = 172).

Factor	*B*	*SE B*	*β*	*t*	*p* value
Constant	57.89	1.61		35.90	<0.001
LoPF identity	0.01	0.07	0.02	0.19	0.852
LoPF self-direction	−0.13	0.06	−0.27	−2.21	0.029*
LoPF empathy	−0.08	0.06	−0.14	−1.52	0.131
LoPF intimacy	−0.15	0.07	−0.21	−2.17	0.031*
sex	3.13	1.29	0.16	2.43	0.016*

Note: *R* = 0.571, *R^2^* = 0.326, B: unstandardized regression coefficient, *SE B*: standard error of unstandardized regression coefficient, *β*: unstandardized regression coefficient, * *p* < 0.05; LoPF = Level of Personality Functioning.

Table 5 shows the results of the multiple linear regression analysis with PF as the dependent variable. The LoPF dimensions explained 6.6% of the variance of PF, and the model was significant with *p* < 0.001. The tests on the assumptions did not indicate multicollinearity of the predictor's identity (VIF = 1.99, *T* = 0.50), self-direction (VIF = 1.97, *T* = 0.51), and intimacy (VIF = 2.08, *T* = 0.48), nor an autocorrelation of the residuals, with a Durbin-Watson value of 1.91. Intimacy showed a significant negative association with the total *Z*-score of PF.

**Table 5.** Results of the multiple linear regression analysis on the dependent variable physical fitness (*N* = 184).

Factor	*B*	*SE B*	*β*	*t*	*P* value
Constant	106.89	1.60		66.82	<0.001
LoPF identity	0.06	0.05	0.11	1.08	0.283
LoPF empathy	−0.06	0.05	−0.12	−1.15	0.252
LoPF intimacy	−0.15	0.07	−0.23	−2.24	0.026*
sex	0.42	1.23	0.03	0.34	0.736

Note: *R* = 0.258, *R^2^* = 0.067, B: unstandardized regression coefficient, *SE B*: standard error of unstandardized regression coefficient, *β*: unstandardized regression coefficient, * *p* < 0.05; LoPF = Level of Personality Functioning

## 4. Discussion

The aim of this study was to examine the associations between personality functioning and both the HRQoL and PF in adolescents. In the simple analyses, the overall LoPF score and the individual dimensions of identity, self-direction, empathy, and intimacy demonstrated significant negative correlations with the HRQoL, thus indicating that greater impairments in personality functioning are associated with a lower quality of life. Similarly, PF showed significant negative correlations with the total LoPF T-score and the dimensions of identity, empathy, and intimacy, thus suggesting that impairments in these areas are linked to reduced PF. Multiple linear regression analyses revealed self-direction and intimacy as negative predictors for the HRQoL and intimacy as a negative predictor for PF.

The findings of the present study on the HRQoL with a mean value of 49.04 are comparable to those of a previous study by Ravens-Sieberer et al. [30], in which adolescents of the same age group achieved an average score of 48.5 for the HRQoL. Regarding mean values for the HRQoL, girls scored well below the gender-specific European normative values – 49.00 for females and 51.12 for males – whereas boys slightly exceeded these normative values, which are indicative of high psychological well-being [30]. This may be attributed to the fact that girls generally exhibit lower HRQoL scores and that their HRQoL was more negatively impacted by the COVID-19 pandemic compared to boys [35],[36]. For this cohort, the mean total Z-score for PF of 103 indicates above-average performance [31] and is comparable to the findings of a study by Cocca et al. [37], which reported a mean *Z*-score of 105 in a sample of more than 900 Austrian secondary school students pre-pandemic. Additionally, the present sample appears to be highly representative, with approximately 25% of adolescents with impairments in personality functioning based on the current state of research – a study by Wagner et al. [38] reported point and lifetime prevalence rates for any psychiatric disorder of 22% and 34%, respectively, in a nationwide study with more than 3600 Austrian adolescents between 10 and 18 years of age.

A multiple linear regression analysis with the HRQoL as the dependent variable identified self-direction and intimacy as significant negative predictors, thus indicating that higher impairment in these dimensions is associated with lower HRQoL. Overall, our findings regarding the significant associations between the HRQoL and the level of LoPF appear to be consistent with previous research, which demonstrated that impairments in personality functioning were associated with a reduced quality of life [39]. In their study conducted with 153 adolescent outpatients aged 14 to 17 at a psychiatric clinic, Korsgaard et al. [39] found that a higher number of personality disorder criteria (assessed using the Structured Interview for DSM-IV Personality) was associated with a reduced quality of life (measured using the Youth Quality of Life Instrument – Research Version).

Notably, the dimension of intimacy appears to play an independent role in both the HRQoL (*β* = −0.21) and physical fitness (*β* = −0.29), thus indicating that higher scores on the intimacy scale – reflecting greater impairment – are associated with lower HRQoL and reduced PF.

In addition to intimacy, self-direction was a significant predictor of the HRQoL. This suggests that both the ability of adolescents to self-regulate and the quality of their interpersonal relationships are closely linked to their perceived quality of life. The negative correlation between self-direction and the HRQoL suggests that deficits in goal-setting and behavioral regulation may impair the quality of life. Similarly, limited intimacy ability, possibly due to reduced social support, appears to negatively affect the quality of life [40].

The present findings align with those of Hofmann et al. [41] and Huang et al. [42], who emphasized the central importance of personality functioning – particularly self-direction – for the mental well-being of young adults. Hofmann et al. [41] showed that individuals with high self-control tend to experience positive emotions more frequently and negative emotions less often. In turn, these regular positive emotions significantly contribute to higher life satisfaction [41]. Additionally, Korsgaard et al. [39] found a negative correlation between the number of fulfilled criteria for personality disorders and self-reported quality of life: the more criteria that were met, the worse adolescents rated their quality of life. Impressively, the four dimensions of LoPF accounted for 30.2% of the variance in the HRQoL in the multiple regression analysis, which, according to Cohen [32], represents a large effect size.

In the regression model with PF as the dependent variable, intimacy emerged as a significant negative predictor, further suggesting that impairments in this domain are associated with lower levels of PF. While there is already some evidence that supports the potential protective role of PF in preventing the onset of mental disorders in adolescents [18],[19], a novel finding of this study is the significant association between personality functioning and PF. Specifically, the total LoPF score and the dimensions of identity, empathy, and intimacy negatively correlated with PF in the simple analysis, even if the effects were only small to moderate. The present findings regarding identity align with the results reported by Timler et al. [43], who found that higher motor competence was associated with a healthier identity (measured using the Assessment of Identity Development in Adolescence (AIDA). However, surprisingly, no significant relationship was found for the self-direction dimension and PF. This contrasts with previous assumptions that self-regulation – a component of self-direction as conceptualized in the LoPF-Q is crucial to maintain physical activity [20]. As shown by Ługowska et al. [44], physical activity contributes to the maintenance or even improvement of PF. Additionally, the literature emphasizes the role of both PF [45] and physical activity for mental well-being in youth [46]. Further supporting the importance of interpersonal skills, findings by Favini et al. [47] suggest that adolescents with higher emotional self-regulation are more physically active. The self-regulation differs from the self-direction dimension in the LoPF, as it was studied in the context of early childhood emotional regulation. Additionally, Vasilopoulos and Ellefson [48] found a positive relationship between physical activity and emotional self-regulation at ages 7, 11, and 14 years, with measured self-regulation encompassing both emotional and behavioral aspects and serving as a foundation for healthy self-direction.

In support of the importance of empathy, Shima et al. [49] found a positive relationship between empathy – measured with the Interpersonal Reactivity Index, which examines non-pathological empathy ability unlike the LoPF-Q and physical activity, likely mediated by increased social engagement, where social interaction acts as a facilitator. Lubans et al. [50] looked at young people from 5 to 18 years old and found that physical activity had positive effects on psychosocial factors such as self-esteem and physical self-concept, which, in turn, correlated with an improved quality of life. Specifically, it was shown that changes in physical self-concept (e.g., perception of one's fitness and body image) were associated with improvements in self-esteem and overall mental health [50]. Zhou et al. [51] highlighted that good physical fitness can boost self-esteem (similar to the identity dimension of the LoPF) and improve social relationships (similar to the intimacy dimension of the LoPF), which could create a positive feedback loop for adolescent development.

Additionally, this study shows a comprehensive connection between overall personality functioning (LoPF total score) and the dimension of intimacy and PF, even if the variance explanation is low at 7%. Previous studies have primarily focused on individual aspects of personality structure and their relationships to physical activity [20],[47]–[49]; to the best of our knowledge, no study has specifically examined personality functioning in relation to PF. Overall, the results suggest that various dimensions of personality functioning are linked to PF, with interpersonal skills seeming to play a central role, while the expected significance of self-direction in this context was not confirmed.

Several countries have already implemented projects to promote mental health within the school context to strengthen emotional and social competencies and to identify mental health issues early. For example, in the United Kingdom, questionnaires and discussions with school psychologists are used to systematically screen for mental health problems [52],[53]. Finland conducts regular screenings and integrates programs for emotional and social development into the school routine [54]. In the United States, schools and universities use psychological tests and programs such as RULER (Recognizing, Understanding, Labeling, Expressing, and Regulating), which aims to promote emotional intelligence through targeted teaching of skills such as recognizing, understanding, naming, expressing, and regulating emotions [55]. Studies show that RULER has positive effects on academic behavior, school climate, and students' social competence [55]. A meta-analysis by Durlak et al. [56] focused on the Social and Emotional Learning (SEL) school intervention program and found significant positive effects across all examined areas, particularly in improving social and emotional skills: positive changes in attitudes, an increase in prosocial behavior, a reduction in behavioral problems and emotional distress, and a significant improvement in academic performance, which increased by about 11 percentile points compared to the control groups. Although Durlak et al. [56] did not explicitly examine the HRQoL or personality functioning, the emotional and social competencies improved by the SEL programs can still be considered indirectly relevant to the student's personal well-being and mental health.

In Austria, the promotion of social and emotional competencies has primarily been embedded in the curriculum for PE at the secondary school level since 2009 [57]. Specifically, cooperation skills, conflict resolution, and emotion regulation are explicitly mentioned as key social competencies [57]. This approach has similarities with the RULER concept but is mainly limited to PE classes. However, a study by Opstoel et al. [58] exclusively focused on PE and organized sports activities, and showed that PE and sports programs played a significant role in promoting social and personal skills in young people, with the methodological implementation and quality of the programs being crucial. Given international findings, it seems sensible to systematically integrate programs into the Austrian school system to promote emotional and social competencies across subjects. A study by Jacobs and Wright [59] showed that fitness games can not only promote physical skills but also important life skills by engaging adolescents in socially responsible behaviors. By combining fitness exercises with social and personal learning goals, students can develop a sense of responsibility, improve their leadership skills, and enhance their self-motivation and subjective health perception [37],[59]. A key point is that the connection between fitness and life skills does not happen automatically, but must be actively supported and reflected upon by the teacher [59]. As a practical implication for PE lessons, the Sport Education Model (SEM) could be applied by teachers, as evidence suggests that the SEM is more need-supportive and fosters intrinsic motivation and prosocial attitudes to a greater extent compared to the skill-drill, direct, and traditional instructional methods commonly used in PE [60]. This knowledge should be included in teacher education alongside psychodynamic foundations of personality development and their connection to school-related quality of life and physical health. In addition to the promotion of social-emotional competencies already established in the curriculum (e.g., through subjects like “social learning” or “social and personal skills”), the integration of physical activity-promoting measures into everyday school life (such as active breaks or enhanced PE) should be implemented. Furthermore, strengthening relationship building between teachers and students – to foster attachment and trust, which are central elements of the “intimacy” dimension – should be emphasized. In addition, school-based health promotion programs that target both physical and mental aspects could be strategically employed to improve an adolescent's HRQoL and, in turn, positively influence their personality development. Additionally, a longitudinal study should examine whether the current integration of social competencies into the curriculum has sustainable effects on emotional well-being, PF, and HRQoL for students in Austria.

Several limitations should be considered when interpreting the results of this study. First, the cross-sectional design does not allow causal conclusions to be drawn. Future longitudinal studies could provide more accurate insights into the predictive value of the personality functioning dimensions on the HRQoL and PF. Second, there is an inherent risk of bias in self-report questionnaires – even if they have been validated, as in the case of the LoPF-Q and KIDSCREEN-10 – due to reduced attention or inaccurate responses [61]. Another limitation is the lack of information on the objective measured physical activity level of the participants, which could potentially have influenced the data. Despite these limitations, the present study provides valuable initial insights, as it is the first to examine the relationships between personality functioning, the HRQoL, and PF in adolescents.

## 5. Conclusions

The results of this study highlight the importance of the self-direction and intimacy scales in relation to the HRQoL, as well as the intimacy dimension in relation to PF. Adolescents who demonstrate strong self-direction and experience high intimacy in their relationships tend to have better health outcomes, such as a better perceived HRQoL and a higher PF. The present results underscore the relevance of personality functioning – particularly intimacy, which reflects the depth and duration of meaningful connections, the capacity and desire for closeness, and mutual regard for others – for both the HRQoL and PF in adolescents. However, longitudinal studies are required to derive actionable recommendations for school health promotion, clarify causal relationships, and further validate these associations. Building a robust data foundation could provide valuable insights for health and intervention planning in the school environment. Therefore, future studies should also focus on the implementation of social competence in the school context to determine whether it serves as a health promotion strategy aimed at strengthening intimacy to improve both mental and physical health in youth.

## Use of AI tools declaration

The authors declare they have not used Artificial Intelligence (AI) tools in the creation of this article.
